# FAM46C is critical for the anti-proliferation and pro-apoptotic effects of norcantharidin in hepatocellular carcinoma cells

**DOI:** 10.1038/s41598-017-00313-6

**Published:** 2017-03-24

**Authors:** Qiao-Yan Zhang, Xiao-Qiang Yue, Yi-Ping Jiang, Ting Han, Hai-Liang Xin

**Affiliations:** 1 Department of Pharmacognosy, School of Pharmacy, Second Military Medical University, Shanghai, 200433 P. R. China; 2 Department of Traditional Chinese Medicine, Changzheng Hospital, Second Military Medical University, Shanghai, 200433 P. R. China

## Abstract

Norcantharidin (NCTD), a demethylated analog of cantharidin derived from Chinese traditional medicine blister beetle, has been currently used as an anticancer drug for various cancers including hepatocellular carcinoma (HCC). In this study, for a more comprehensive understanding of the targets of NCTD in HCC, next-generation RNA-Seq was utilized. We revealed that the expression of FAM46C, which has been reported as a tumor suppressor for multiple myeloma, was enhanced after NCTD treatment. Re-analysis of TCGA (The Cancer Genome Atlas) LIHC (liver hepatocellular carcinoma) dataset demonstrated that FAM46C expression was significantly lower in HCC tissues than in normal liver tissues. NCTD injection or FAM46C overexpression could mitigate diethylnitrosamine (DEN)-initiated HCC in mice. Ectopic expression of FAM46C in two HCC cell lines, SMCC-7721 and SK-Hep-1, significantly repressed cell proliferation, and increased cells population in G2/M phase and cell apoptotic rate. We also found that FAM46C overexpression caused a notable decrease in Ras expression, MEK1/2 phosphorylation and ERK1/2 phosphorylation. More importantly, FAM46C knockdown significantly weakened the biological effects of NCTD on HCC cells, which suggested NCTD exerted the anticancer functions partially through up-regulating FAM46C. In conclusion, FAM46C, a tumor suppressor for HCC, is important for the anti-proliferation and proapoptotic effects of NCTD.

## Introduction

Hepatocellular carcinoma (HCC) is one of the most common cancers in the world and remains one of the leading causes of cancer mortality^1,2^. Most HCC patients were diagnosed at advanced stage, and only 30% were surgically resectable^3^. Patients with advanced HCC had limited treatment options, such as radiofrequency ablation, selective radiotherapy, selective chemotherapy, systemic chemotherapy and transarterial chemoembolization^4^. Thus, the 5-year survival rate for HCC patients is less than 20%^2^.

Norcantharidin (NCTD) is a demethylated analog of cantharidin derived from the dried body of Chinese traditional medicine blister beetle (Mylabris phalerata Pallas)^5^. In China, NCTD has been used to treat patients with HCC, breast cancer, colon cancer, leukemia, etc. for many years^6^. Previous studies have demonstrated the anti-proliferation and pro-apoptotic effects of NCTD on numerous tumor cell lines *in vitro* and tumor models *in vivo* ^7–21^. Several mechanisms responsible for pro-apoptotic effects of NCTD have been explored. NCTD can modulate the expression of Bcl-2 superfamily member proteins^8,9,15^ and caspase activity^8,20^. NCTD-induced apoptosis in glioblastoma cells^18^ and oral cancer cells^15^ is dependent on p53. NCTD exerts pro-apoptosis effects in colorectal cancer cells via the activation of the CD95 receptor/ligand system^17^. In addition, the activities of mitogen-activated protein kinase (MAPK) family members, such as extracellular signal-regulated kinase (ERK), p38MAPK or c-Jun N-terminal kinase (JNK) have been found involved in NCTD-induced apoptogenesis in breast cancer cells^12^, colon cancer^10^ and glioma cells^14^. In HCC cells, the modulation of NCTD on cell cycle-related proteins and kinase activity may contribute to NCTD-induced cell cycle arrest^8^.

The current study confirmed the anti-proliferation effects of NCTD on HCC cells through inducing cell-cycle arrest and cell apoptosis. For a more comprehensive understanding of the targets of NCTD in HCC, next-generation RNA-Seq was utilized. FAM46C, which is a non-canonical poly(A) polymerase and may regulate gene expression during cell differentiation^22^, was found as a potential target of NCTD. FAM46C expression was significantly decreased in HCC tissues when compared with normal liver tissues. NCTD injection or FAM46C overexpression could mitigate diethylnitrosamine (DEN)-initiated HCC in mice. Then *in vitro* experiments indicated the critical role of FAM46C in the anti-proliferation effects of NCTD on HCC cells.

## Results

### Effect of NCTD on the proliferation, cell cycle distribution and apoptosis of HCC cells

**Figure 1:**
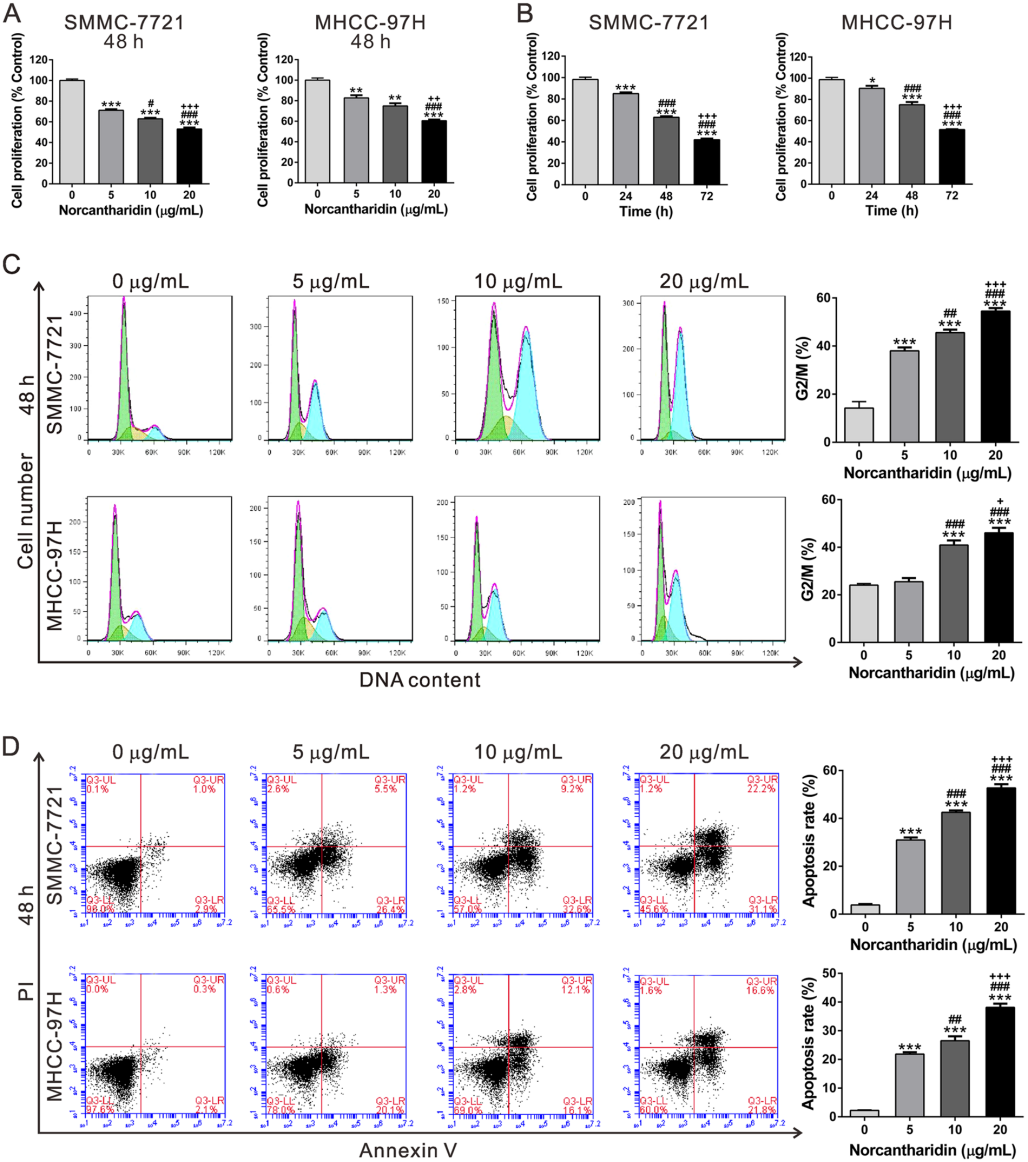
Effects of NCTD on cell proliferation and apoptosis of SMCC-7721 and MHCC-97H cells. (**A**) SMCC-7721 and MHCC-97H cells were treated with DMSO or NCTD (5, 10 and 20 µg/mL) for 48 h. CCK-8 assay was carried out to assess cell proliferation. The relative cell proliferation was defined as the percentage of cells treated with DMSO (% Control). **P < 0.01, ***P < 0.001 as compared with DMSO group; ^#^ *P* < 0.05, ^###^ *P* < 0.001 as compared with 5 µg/mL NCTD-treated group; ^++^ *P* < 0.01, ^+++^ *P* < 0.001 as compared with 10 µg/mL NCTD-treated group. (**B**) The cells were treated by 10 μg/mL NCTD for 24, 48 and 72 h. At the end of incubation, CCK-8 assay was carried out to assess cell proliferation. The relative cell proliferation was expressed as the percentage of OD_450_ compared with that of the control (% Control). *P < 0.05, ***P < 0.001 as compared with 0 h; ^###^ *P* < 0.001 as compared with 24 h; ^+++^ *P* < 0.001 as compared with 48 h. (**C,D**) SMCC-7721 and MHCC-97H cells were treated with DMSO or NCTD for 48 h. Cell cycle (**C**) distribution was assessed by PI staining and flow cytometric analysis. Cell percentages in G2/M phase were shown here. Cell apoptosis (**D**) was evaluated by Annexin V-FITC/PI staining followed by flow cytometric analysis. Cells in the lower right quadrant are Annexin V-positive and PI-negative staining, representing the early apoptotic cells. ***P < 0.001 as compared with DMSO group; ^##^ *P* < 0.01, ^###^ *P* < 0.001 as compared with 5 µg/mL NCTD-treated group; ^+++^ *P* < 0.001 as compared with 10 µg/mL NCTD-treated group. All experiments shown were performed independently at least three times.

In order to investigate the effect of NCTD on HCC cell proliferation, CCK-8 assay was performed. SMCC-7721 and MHCC-97H cells were exposed to increasing doses of NCTD (5, 10 and 20 µg/mL) for 48 h. NCTD was dissolved in DMSO, thus DMSO was served as a negative control. Figure 1A showed that 48 h of NCTD treatment significantly decreased HCC cell growth in a dose-dependent manner. CCK-8 assay was also carried out on SMCC-7721 and MHCC-97H cells treated with 10 µg/mL NCTD for 0, 24, 48 and 72 h. The results showed that NCTD treatment time dependently reduced the proliferation of both HCC cell lines (Fig. 1B).

We further investigated the effect of NCTD on cell cycle distribution and cell apoptosis. Our results showed that treatment of SMCC-7721 and MHCC-97H cells with 10 µg/mL NCTD for 48 h significantly increased cells in G2/M phase of cell cycle (Fig. 1C) and the incidence of apoptosis (Fig. 1D) in a dose-dependent manner.

### FAM46C expression is elevated by NCTD treatment

**Figure 2:**
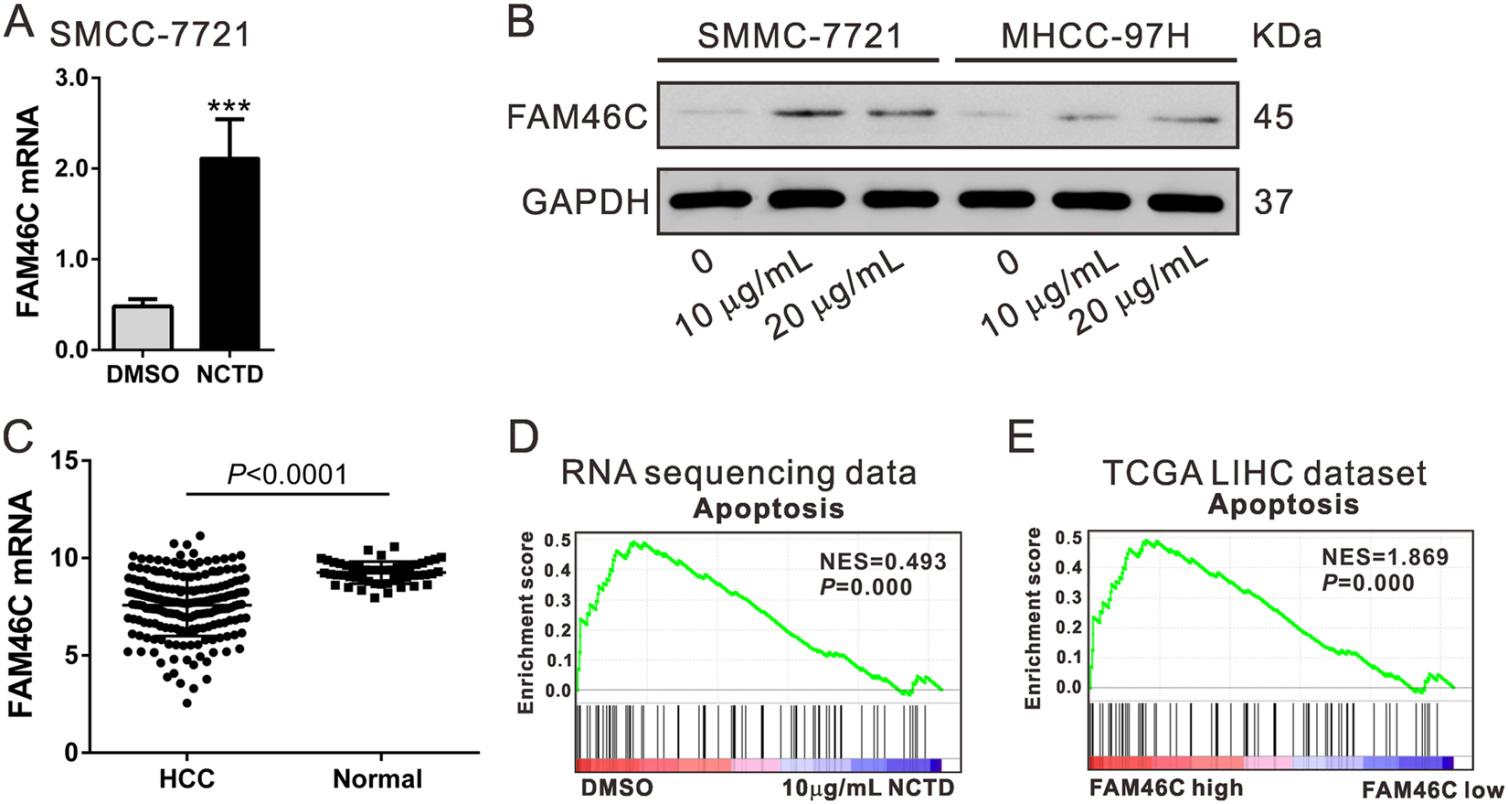
Effects of NCTD on FAM46C expression. (**A**) RNA-sequencing data showed that NCTD significantly induced FAM46C mRNA expression. (**B**) SMCC-7721 and MHCC-97H cells were treated with DMSO or NCTD (10 and 20 µg/mL) for 48 h. The protein level of FAM46C was significantly induced by NCTD treatment. (**C**) FAM46C was down-regulated in HCC tissues (n = 191) compared to the normal tissues (n = 50) in TCGA LIHC dataset. (**D**) GSEA revealed that the cell apoptosis pathway was closely related with NCTD treatment. Black bars indicated individual genes in rank order. NES, normalized enrichment score. (**E**) GSEA was performed using TCGA LIHC dataset. The cell apoptosis pathway was strongly associated with FAM46C expression.

The above experiments on cell proliferation, cell cycle and apoptosis showed that SMCC-7721 cells were more sensitive to NCTD than MHCC-97H cells. To explore how NCTD exerted cytotoxic effects on HCC cells, a total of 6 RNA samples, collected from three biological replicates of DMSO or NCTD (10 µg/mL)-treated SMCC-7721 cells were subjected to RNA sequencing. We identified 1,435 up-regulated (Table S1) and 1,435 down-regulated genes (Table S2) in SMCC-7721 cells treated with NCTD by using fold change > 2 and P-value < 0.05 as cut-off. Among the most significantly altered genes, FAM46C was significantly induced by NCTD treatment (Fig. 2A). Moreover, Western blotting analysis showed that NCTD treatment (10 and 20 µg/mL) significantly increased the protein level of FAM46C (Fig. 2B). FAM46C has been recently reported as a tumor suppressor for multiple myeloma^23–27^. By re-analyzing expression data of TCGA (The Cancer Genome Atlas) LIHC (liver hepatocellular carcinoma) cohort, we found that FAM46C expression was significantly lower in HCC tissues than in the normal liver tissues (*P* < 0.0001, Fig. 2C), suggesting that FAM46C was a potential target for NCTD in HCC. Thus, we chose FAM46C for further investigation.

To determine which biological pathways were involved in NCTD treatment and to elucidate whether FAM46C was involved in HCC pathogenesis, GSEA was further performed on the RNA sequencing data and LIHC cohort from TCGA, respectively. GSEA results showed that cell apoptosis pathway was associated with NCTD treatment (Fig. 2D) and FAM46C expression (Fig. 2E). These data suggested that FAM46C may be involved in the effects of NCTD on HCC cells.

### NCTD injection and FAM46C overexpression attenuates DEN-initiated HCC formation in mice

**Figure 3:**
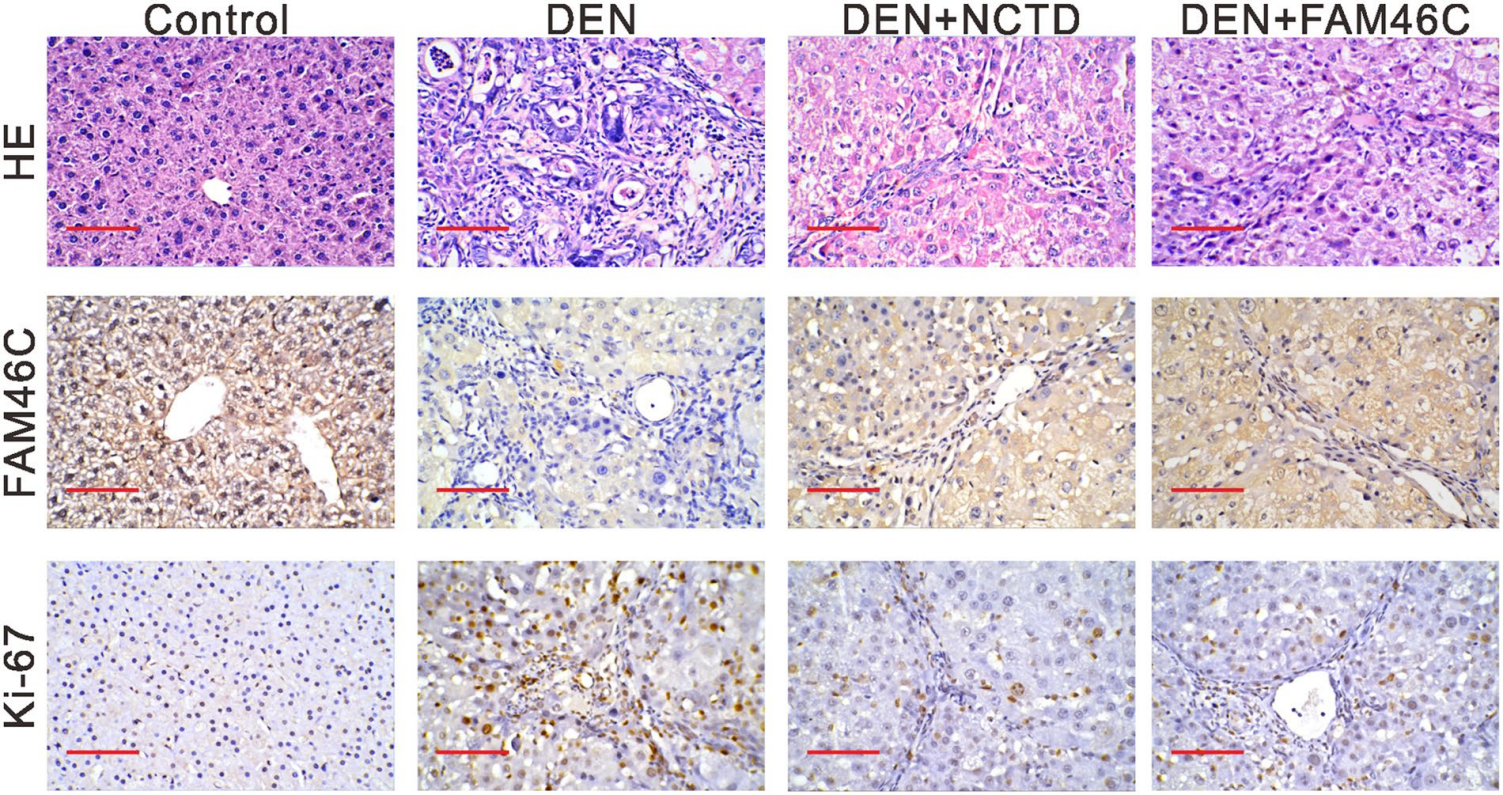
Pathological changes of liver in 4 groups mice (n = 8 per group). Light microscopy of HE staining, and IHC staining of FAM46C and Ki-67 (200x magnification) in Control group, DEN group, DEN+NCTD group and DEN+FAM46C group.

To confirm the function of NCTD and FAM46C *in vivo*, mice were injected with DEN to initiated HCC, and then treated with NCTD or mouse FAM46C lentivirus. The liver histological changes were compared by using Hematoxylin-Eosin (HE) staining (Fig. 3). Severe hepatic fibrosis, fatty degeneration and bridging necrosis were observed in DEN group, indicating the formation of HCC. NCTD or FAM46C lentivirus treatment attenuated the extent of histological changes. As indicated by immunohistochemical (IHC) staining, FAM46C expression in the livers was reduced by DEN treatment, while additional NCTD or FAM46C lentivirus treatment increased FAM46C expression. Cell proliferation was also determined by Ki-67 IHC staining. Ki-67-positive signal was weak in the control sections, but strong and widely distributed throughout the liver sections of DEN group. In the groups with additional NCTD or FAM46C lentivirus treatment, Ki-67-positive signal was decreased compared with DEN group. These data suggested that NCTD injection and FAM46C overexpression could mitigate HCC in mice.

### FAM46C overexpression inhibits HCC cell proliferation

**Figure 4:**
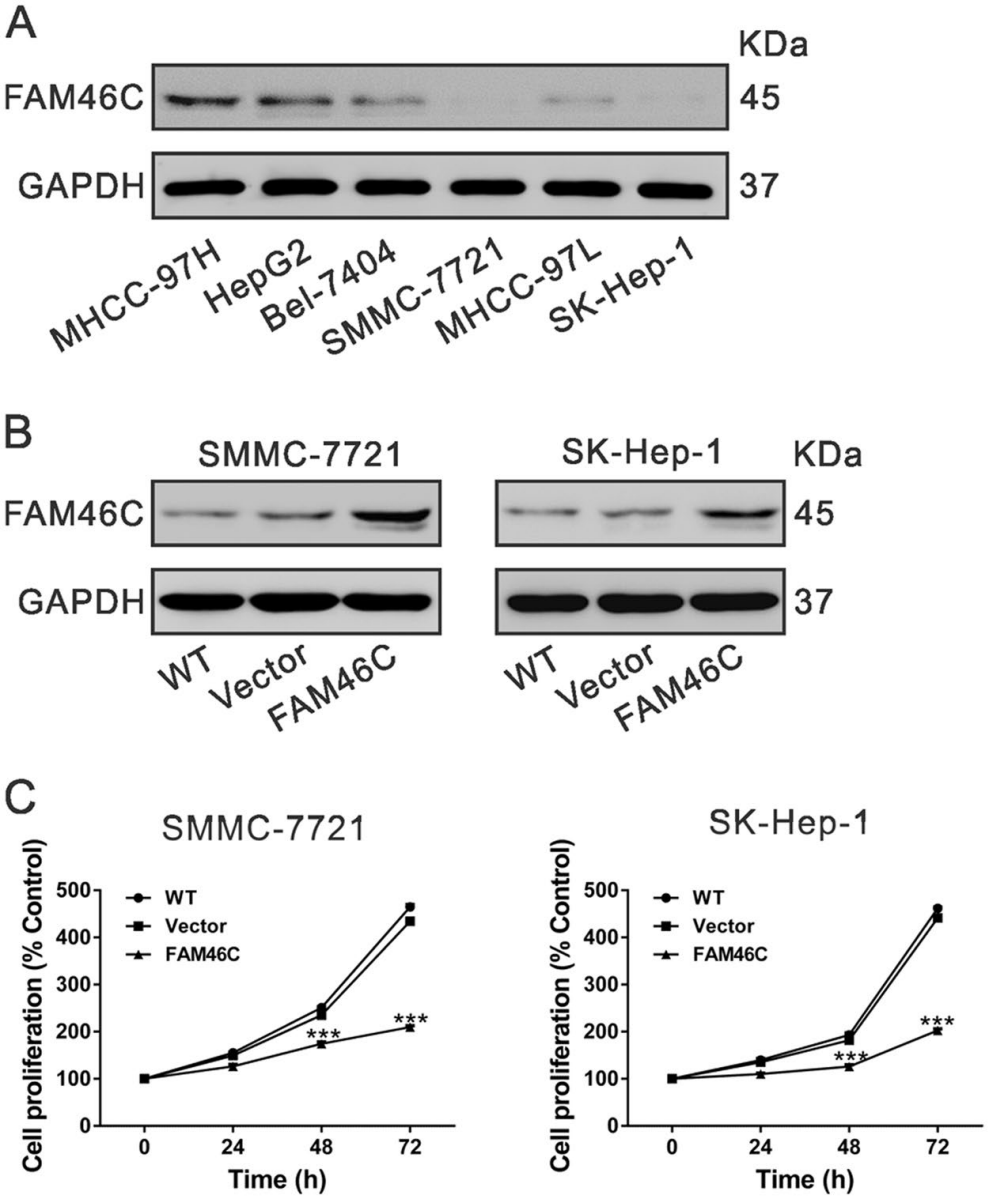
FAM46C overexpression inhibited HCC cell proliferation. (**A**) Expression of FAM46C in 6 HCC cell lines as determined by Western blotting. (**B**) Ectopic expression of FAM46C in SMCC-7721 and SK-Hep-1 cells was examined by Western blotting. (**C**) Ectopic expression of FAM46C significantly decreased cell proliferation as determined by CCK-8 assay. All experiments shown were performed independently at least three times. ***P < 0.001 as compared with Vector virus-infected cells.

FAM46C expression was then estimated in 6 HCC cell lines by western blotting. Two cell lines, SMCC-7721 and SK-Hep-1, showed lower level of FAM46C (Fig. 4A).To investigate the function of FAM46C in HCC cells, SMCC-7721 and SK-Hep-1 cells were infected with vector control or FAM46C lentivirus. The ectopic expression of FAM46C in both cells was confirmed by Western blotting (Fig. 4B).

To determine the effect of FAM46C on cell proliferation, we monitored the proliferation rate of HCC cells for 3 days by CCK-8 assay. We observed that SMCC-7721 and SK-Hep-1 cells overexpressed FAM46C exhibited a significantly slower growing phenotype than corresponding control cells (WT and Vector cells, Fig. 4C). These results indicated that FAM46C exerted growth inhibitory effects on HCC cells.

### FAM46C overexpression induces G2/M phase arrest and cell apoptosis

**Figure 5:**
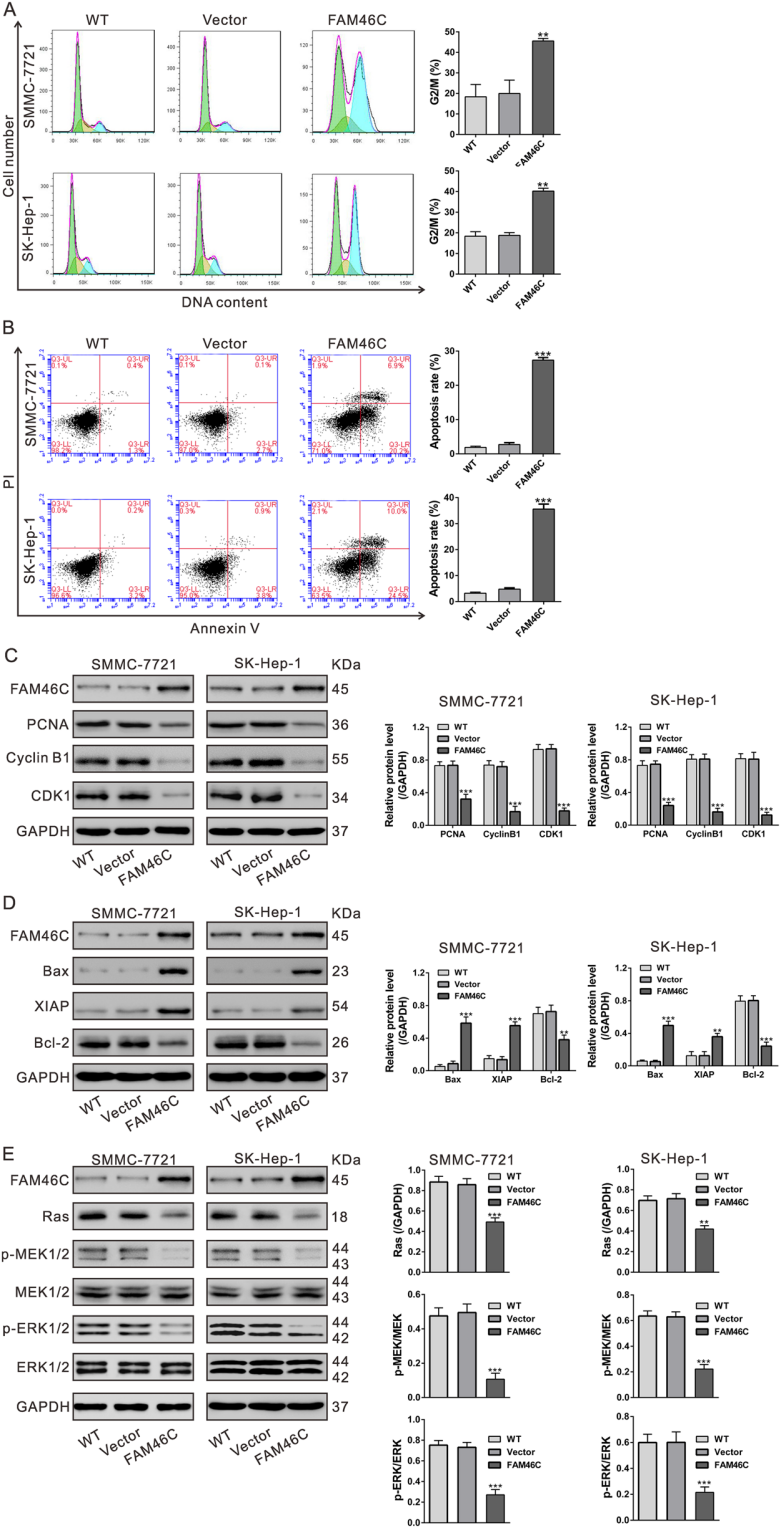
FAM46C overexpression resulted in G2/M phase arrest (**A**) and a significant increase of cell apoptosis (**B**) as determined by flow cytometry analyses at 48 h after viral infection. ***P < 0.001 as compared with Vector virus-infected cells. (**C**) Protein levels of FAM46C and cell growth-related factors (PCNA, Cyclin B1 and CDK1) were evaluated by Western blotting. (**D**) Protein levels of FAM46C and apoptotic factors (Bcl-2, Bax and XIAP) were assessed. (**E**) The levels of Ras, p-MEK1/2, MEK, p-ERK1/2 and ERK1/2 were evaluated. All experiments shown were performed independently at least three times. **P < 0.01 and ***P < 0.001 as compared with Vector virus-infected cells.

Next, flow cytometric analysis was performed to further examine whether FAM46C affected the proliferation of HCC cells by altering cell-cycle progression and cell apoptosis. Figure 5A revealed that cell-cycle progression of HCC cells overexpressed FAM46C was significantly accumulated at the G2/M phase compared to corresponding control cells (WT and Vector). Moreover, overexpression of FAM46C remarkably induced the early apoptosis of SMCC-7721 and SK-Hep-1 cells (Fig. 5B and Fig. S1).

### Mechanisms of FAM46C exerts its function

Western blotting was performed to analyze the expression of cell growth and apoptosis-related proteins (Fig. 5C and D). In accordance with the above functional experiments, ectopic expression of FAM46C significantly down-regulated cell growth (PCNA^28^, Cyclin B1 and CDK1^29^) and anti-apoptosis (Bcl-2^30,31^) related factors in both SMCC-7721 and SK-Hep-1 cell lines compared with the control cells, while remarkably up-regulated the apoptosis factor (Bax^32^ and XIAP^33^). Complementary results were obtained in MHCC-97H cells with FAM46C knockdown (Fig. S2).

Ras/MEK/ERK pathway has been suggested to promote cell proliferation and suppressed cell apoptosis^34,35^. We then assessed Ras, p-MEK1/2, MEK1/2, p-ERK1/2 and ERK1/2 in both SMCC-7721 and SK-Hep-1 cells. Over-expression of FAM46C dramatically decreased Ras levels, as well as the phosphorylation status of MEK1/2 and ERK1/2 (Fig. 5E). Complementary results were also obtained in MHCC-97H cells with FAM46C knockdown (Fig. S2).

### FAM46C is critical for the anti-cell growth effects of NCTD on HCC cells

**Figure 6:**
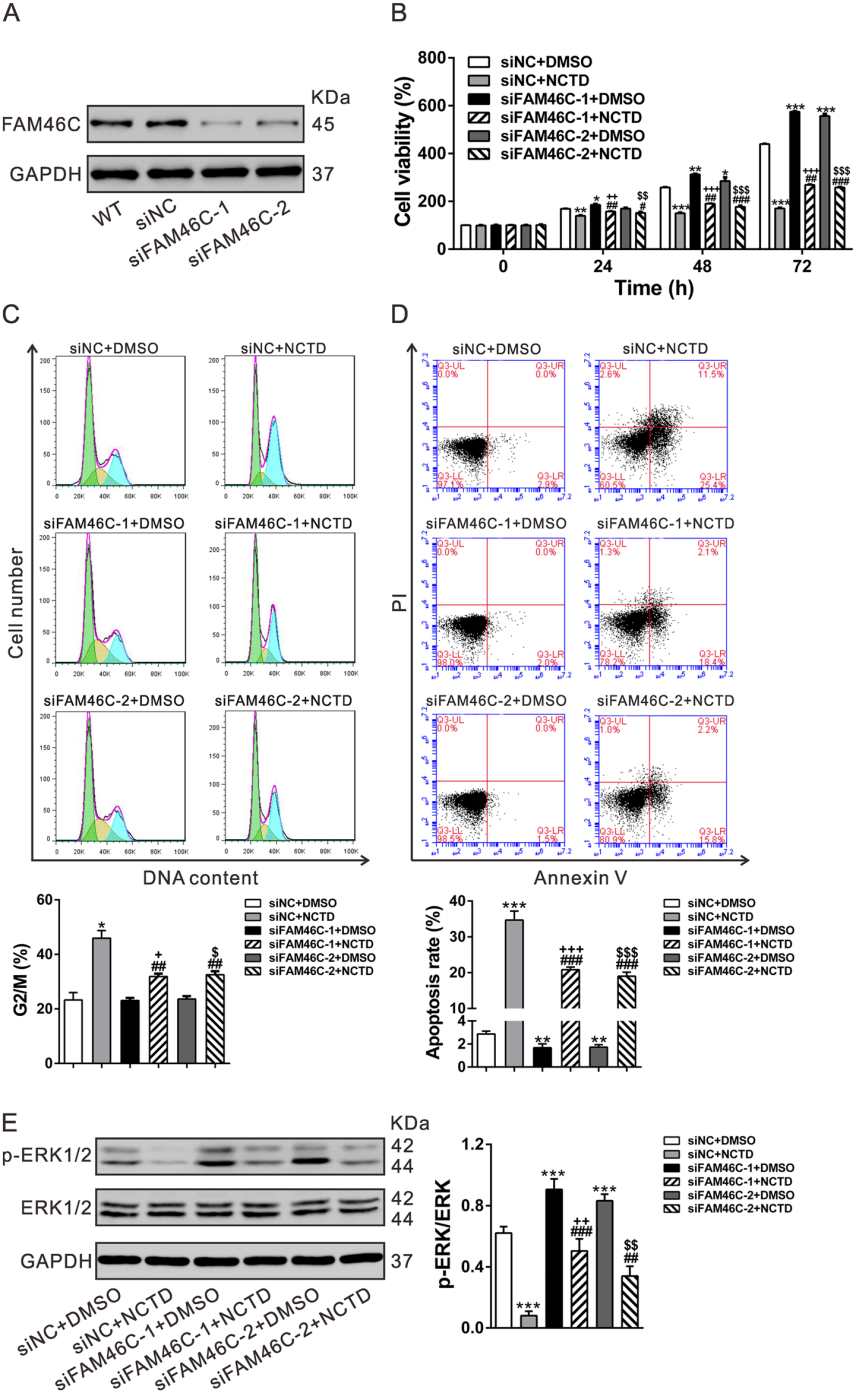
FAM46C is critical for the anti-cell growth effects of NCTD on HCC cells. (**A**) MHCC-97H cells were transfected with siNC, siFAM46C-1 or siFAM46C-2. At 48 h after transfection, FAM46C protein expression was detected by Western blotting. (**B,C,D**) MHCC-97H cells were divided into 6 groups: siNC+DMSO, siNC+NCTD (10 μg/mL), siFAM46C-1+DMSO, siFAM46C-1+NCTD (10 μg/mL), siFAM46C-2+DMSO and siFAM46C-2+NCTD (10 μg/mL). Cell proliferation (B) was evaluated by CCK-8 assay at indicated time points. At 48 h after treatment, cell percentages in G2/M phase (**C**) and cell apoptosis (**D**) were detected by flow cytometric analysis. (**E**) Western blot analysis of ERK phosphorylation at 48 h after treatment. All experiments shown were performed independently at least three times. *P < 0.05, **P < 0.01, ***P < 0.001 as compared with siNC+DMSO; ^##^ *P* < 0.01, ^###^ *P* < 0.001 as compared with siNC+NCTD; ^+^ *P* < 0.001, ^++^ *P* < 0.05,^+++^ *P* < 0.001 as compared with siFAM46C-1+NCTD; ^$^ *P* < 0.001, ^$$^ *P* < 0.05, ^$$$^ *P* < 0.001 as compared with siFAM46C-2+NCTD.

Moreover, to further explore the association between NCTD and FAM46C, FAM46C expression was suppressed by siRNA transfection. As shown in Fig. 6A, FAM46 siRNA (siFAM46C-1 or siFAM46C-2) notably decreased its protein expression in MHCC-97H cells compared with control siRNA (siNC).

MHCC-97H cells were then divided into 6 groups: Group 1, cells treated with siNC and DMSO; Group 2, cells treated with siNC and 10 μg/mL NCTD; Group 3, cells treated with siFAM46C-1 and DMSO; and Group 4, cells treated with siFAM46C-1 and 10 μg/mL NCTD; Group 3, cells treated with siFAM46C-2 and DMSO; and Group 4, cells treated with siFAM46C-2 and 10 μg/mL NCTD. After 24 h of treatment, CCK-8 assay showed that silencing of FAM46C could partially abrogate the proliferation-inhibitory effects of NCTD on MHCC-97H cells (Fig. 6B). Further, silencing of FAM46C could partially counteract the inducing action of NCTD on G2/M (Fig. 6C) and suppressing cell apoptosis (Fig. 6D).

We then detected phosphorylation status of ERK1/2 (Fig. 6E). ERK1/2 activity was decreased by NCTD treatment, and partially rescued by FAM46C knockdown. These findings clearly indicated that NCTD exerted its function via regulating FAM46C and ERK1/2 signaling.

## Discussion

The anti-proliferation effects of norcantharidin (NCTD) on HCC cells have been studied *in vitro* ^8^ and *in vivo* ^11^. Previous studies indicated that NCTD may exert its functions by regulating MAPKs signaling pathways in other cancer cells^10,12,14^. Here, we found that FAM46C expression was enhanced by NCTD treatment. Its overexpression inactivated MAPK/ERK signaling and inhibited the proliferation of HCC cell lines.

Firstly, we confirmed that NCTD treatment significantly decreased the proliferation of SMCC-7721 and MHCC-97H cells via inducing G2/M phase arrest and cell apoptosis (Fig. 1), which was in accordance with the previous study^8^. RNA sequencing results showed that FAM46C was increased after NCTD treatment (Fig. 2A). FAM46C was down-regulated in HCC tissues compared with normal liver tissues in TCGA LIHC dataset. These data suggested that FAM46C may be involved in the HCC pathogenesis and a target for NCTD. It was noteworthy that SMCC-7721 was more sensitive to NCTD treatment, which may due to the relative lower FAM46C expression in SMCC-7721 cells than in MHCC-97H cells (Fig. 4A).

FAM46C is a non-canonical poly(A) polymerase^22^ and have been reported as a potential marker and a tumor suppressor for multiple myeloma^23–27^. Point mutation of FAM46C has been found in patients with various tumors including HCC^22^, suggesting its involvement in this malignancy. In the present study, we examined the functions of FAM46C in HCC cells. Ectopic expression of FAM46C in HCC cells with lower expression of FAM46C could repress cell proliferation (Fig. 4), and induce G2/M phase arrest and cell apoptosis (Fig. 5), whereas FAM46C knockdown resulted in inverse effects on HCC cells with lower expression of FAM46C (Fig. 6). Our data indicated for the first time that FAM46C may be a potential tumor suppressor in HCC.

Moreover, we tried to explore the molecular mechanism by which FAM46C functions as a tumor suppressor in HCC. ERK belongs to the mitogen-activated protein kinase (MAPK) family, activated by mitogenic stimuli, is critical for proliferation and survival^34,35^. Activated ERK has been associated with the expression of key modulators for G2/M arrest, such as Cyclin B1 and CDK1^29^. ERK activity also has been linked to the downregulation of anti-apoptotic protein, Bcl-2^30,31^, and upregulation of proappoptotic proteins, Bax^32^ and XIAP^33^. In the present study, FAM46C overexpression caused a remarkable decrease in ERK activation, expression of Cyclin B1, CDK1 and Bcl-2 (Fig. 5), and a remarkable increase of Bax expression. In addition, the upstream activator of ERK, Ras and MEK, was changed in concordance with ERK inactivation. These results suggested that FAM46C may exerted its anti-proliferation and proapoptotic functions via Ras/MEK/ERK signaling pathways although more studies are necessary. Moreover, NCTD treatment (10 and 20 µg/mL) significantly induced FAM46C protein expression (Fig. 2B) and had the same effects on the changes of the above detected molecules as FAM46C overexpression (Fig. S3). These data suggest that FAM46C is a potential target for NCTD.

To further investigate the association between NCTD and FAM46C, FAM46C expression was suppressed by siRNA transfection in MHCC-97H cells. The biological effects of NCTD on HCC cells were significantly weakened by FAM46C knockdown (Fig. 6), which suggested NCTD exerted the anticancer functions partially through up-regulating FAM46C. Moreover, it was reported that NCTD activated ERK in breast cancer cells^12^, while other studies on colon cancer^10^ and glioma cells^14^ found that NCTD could inhibited ERK phosphorylation. We found that ERK activity was decreased by NCTD treatment, and partially rescued by FAM46C knockdown. Our findings and the previous studies suggested the regulation of NCTD on ERK phosphorylation is cell-type dependent. These findings clearly indicated that NCTD exerted its function via regulating FAM46C and ERK1/2 signaling.

In conclusion, FAM46C expression was lower in HCC tissues than in normal liver tissues. FAM46C may serve as a tumor suppressor during hepatocellular tumorigenesis. FAM46C is important for the anti-proliferation and proapoptotic effects of NCTD on HCC.

## Materials and Methods

### Cell culture

The human cell lines, HEK293T, MHCC-97H, HepG2, Bel-7404, SMMC-7721, MHCC-97L and SK-Hep-1, were obtained from Chinese Type Culture Collection, Chinese Academy of Sciences (Shanghai, China) and cultured in a humidified atmosphere at 37 °C with 5% CO_2_. All cell lines were cultured in Dulbecco’s modified Eagle’s medium (DMEM; Life Technologies, Carlsbad, CA, USA) containing 10% fetal bovine serum, 100 μg/ml streptomycin and penicillin (Life Technologies).

### NCTD treatment

SMMC-7721 and MHCC-97H cells were exposed to various concentrations of NCTD (dissolved in DMSO; 5, 10 and 20 μg/mL; Sigma, St. Louis, MO, USA) or DMSO. Cell proliferation, cell cycle distribution and cell apoptosis was then assessed by Cell Counting Kit-8 (CCK-8) Assay (Dojindo Lab, Kumamoto, Japan), propidium iodide (PI) staining and Annexin V/PI staining kit (BD Biosciences, San Jose, CA, USA), respectively.

CCK-8 assay was performed in 96-well culture plates as previously described^22^. At indicated time after treatment, CCK-8 solution was added to each well and incubated for an additional 1 h. The absorbance was measured at 450 nm on a microplate reader (Bio-Rad, Hercules, CA, USA). The relative cell proliferation was defined as the percentage of cells treated with DMSO.

Cells plated in 6-well plates were collected at 48 h after treatment. For cell cycle analysis, cells were fixed with ice-cold 70% ethanol at −20 °C overnight, washed with PBS, and then incubated with 50 μg/ml PI (Sigma) and 100 U/ml ribonuclease A (Sigma) in the dark for 30 min. DNA content was analyzed using a flow cytometer (BD Biosciences). For cell apoptosis analysis, cells were collected, washed with PBS, and double-labeled with Annexin V-FITC and PI (BD Biosciences) followed by flow cytometric analysis. Cells in the lower right quadrant are Annexin V-positive and PI-negative staining, representing the early apoptotic cells.

### RNA extraction and sequencing

SMMC-7721 cells were exposed to 10 μg/mL NCTD or DMSO for 24 h, collected and then placed in the Trizol reagent (Invitrogen, Carlsbad, CA, USA). RNA was isolated as per manufacturer’s instructions and the integrity of RNA was assessed by denaturing formaldehyde gel electrophoresis. Libraries for RNA-sequencing were prepared with Illumina’s TruSeq Sample Preparation Kit. Next-generation sequencing was carried out using the Illumina Genome Analyzer with the standard protocol (Illumina, San Diego, CA). The sequence data have been deposited in NCBI’s Sequence Read Archive database (http://www.ncbi.nlm.nih.gov/sra, AC: SRP075227).

### Bioinformatics analysis

Gene expression data were obtained at The Cancer Genome Atlas website (TCGA, https://tcga-data.nci.nih.gov/tcga/) for the LIHC (liver hepatocellular carcinoma) projects, and FAM46C expression was analyzed on 191 HCC tissues and 50 normal liver tissues.

To determine which biological pathways were involved in NCTD treatment and to elucidate whether FAM46C was involved in HCC pathogenesis, Gene set enrichment analysis (GSEA) was performed based on our sequencing data and TCGA LIHC dataset as previously described^36,37^.

### Western blotting

Cells were lysed with radioimmunoprecipitation assay buffer (Beyotime, Shanghai, China). After the protein concentration was determined with BCA protein assay kit (Thermo Fisher Scientific), equal protein aliquots (25 μg per lane) were resolved on SDS gel electrophoresis and Western blotting assay was performed with standard protocol. The expression of target proteins was detected by using enhanced chemiluminescence system (Bio-Rad, Richmond, CA, USA) and quantified by densitometry with Image J software (http://rsb.info.nih.gov/ij/, Bethesda, MD, USA). Protein expression was normalized by the loading control (GAPDH) expression. Antibodies against FAM46C (ab169699), CDK1 (ab32384), XIAP (ab2541) and Ras (ab52939) were purchased from Abcam (Cambridge, MA, USA). Anti-Bcl-2 (sc-492) and anti-Bax (sc-493) were from Santa Cruz Biotech. (Santa Cruz, CA, USA). Antibodies against Cyclin B1 (#4135), PCNA (#13110), p-MEK1/2 (#8211), MEK1/2 (#9122), p-ERK1/2 (#4376), ERK (#4695) and GAPDH (#5174) were purchased from Cell Signaling (Danvers, MA, USA).

### Construction of lentivial vectors and lentiviral production

The full length human FAM46C or mouse FAM46C CDNA was inserted into GV303 (Genechem, Shanghai, China), a lentiviral vector carrying Ubi promoter and SV40-enhanced green fluorescent protein (EGFP) as previously described﻿^38^. Lentiviral constructs of GV303 empty vector, GV303-human FAM46C or GV303-mouse FAM46C were then cotransfected with pHelper1.0 and pHelper 2.0 (Genechem) into HEK293T cells by using lipofectamine 2000 (Invitrogen) following the manufacture’s instruction. After 48–72 h, viral supernatant was harvested and filtered through 0.45-μm filter. The lentiviral titer was determined by counting GFP-positive cells. SMMC-7721 and SK-Hep-1 cells were infected with human FAM46C lentivirus or Vector lentivirus at a multiplicity of infection (MOI) of 50 in the presence of polybrene (Sigma, 6 μg/mL). About 48 h later, FAM46C expression was analyzed by Western blotting.

### RNA interference

To knock down FAM46C expression, the specific siRNA fragments of FAM46C (siFAM46C-1, 5′-GGACGAGGCAACUUUCCAAUU-3′ and siFAM46C-2, 5′-GCAACUUCAGCAACUACUAUU-3′) and control siRNA (5′-CCUAAGGUUAAGUCGCCCUCG-3′) were designed and synthesized by Genepharma Co., Ltd (Shanghai, China). For transfection, MHCC-97H cells were seeded and allowed to reach 70–80% confluence. Then, cells were transfected with 2 μg/ml siRNA with lipofectamine 2000 (Invitrogen). Approximately 24 h later, the transfection efficiency was analyzed by Western blotting.

### Animal experiments

All animal experiments were approved and performed according to the guidelines of ethical review boards at Second Military Medical University. Male C57BL/6 J mice (6–8 weeks old) obtained from Shanghai Experimental Animal Center (Shanghai, China) were housed in a 12-h light-dark cycle with free access to sterilized tap water and chow diet. The animal experiments were in accordance with guidelines of the Institutional Animal Care and Use Committee. Diethylnitrosamine (DEN) was obtained from Sigma. Forty mice were divided into 4 groups, included: Control group, DEN group, DEN+NCTD group and DEN+FAM46C group (n = 10 per group). The mice in DEN group were intraperitoneal (ip) given DEN at doses of 100 mg/kg, and 50 mg/kg in the following week. In addition to the processing of DEN group, the mice in DEN+NCTD group were ip injected with NCTD (2 mg/kg) daily on 4 weeks after the first injection of DEN; and the mice in DEN+FAM46C group were intravenous injected with mouse FAM46C lentivirus (1 × 10^7^ pfu per mouse) weekly on 4 weeks after the first injection of DEN. Untreated mice were used as controls. After another 3 weeks, mice were killed under sodium pentobarbital anesthesia. Liver samples were collected, fixed in 10% formalin, embedded in paraffin, and then cut into 4-μm slices. HE staining was performed to detect the liver histological changes, while IHC staining with anti-FAM46C (Novus Biologicals, Inc.; Littleton, CO, USA) or anti-Ki-67 (Abcam) was carried out to detected FAM46C expression or cell proliferation.

### Statistical analysis

All numerical results were analyzed by GraphPad Prism software (version 6.0, San Diego, CA, USA) and shown as means ± SD. The Student’s t test was performed for comparisons between two groups, while one-way analysis of variance (ANOVA) followed by Sidak’s test was performed for multiple comparisons. *P* < 0.05 was considered as statistically significant.

## Electronic supplementary material

Supplementary Information

Supplementary Table S1

Supplementary Table S2
