# Author Correction: FAM46C is critical for the anti-proliferation and pro-apoptotic effects of norcantharidin in hepatocellular carcinoma cells

**DOI:** 10.1038/s41598-017-17244-x

**Published:** 2017-12-11

**Authors:** Qiao-Yan Zhang, Xiao-Qiang Yue, Yi-Ping Jiang, Ting Han, Hai-Liang Xin

**Affiliations:** 1 Department of Pharmacognosy, School of Pharmacy, Second Military Medical University, Shanghai, 200433 P. R. China; 2 Department of Traditional Chinese Medicine, Changzheng Hospital, Second Military Medical University, Shanghai, 200433 P. R. China

*Scientific Reports* **7**:396; doi:10.1038/s41598-017-00313-6; Article published online 24 March 2017

Two supplementary tables were omitted from the original version of this article. This has now been corrected in the HTML version of the Article; the PDF version was correct at the time of publication.

