# Supplementary material for: FAM46C is critical for the anti-proliferation and pro-apoptotic effects of norcantharidin in hepatocellular carcinoma cells
Source: Sci Rep. 2017 Mar 24;7:396. doi: 10.1038/s41598-017-00313-6 (PMC5428258; doi:10.1038/s41598-017-00313-6)
Supplement: Supplementary file 1 — Supplementary Information [file 41598_2017_313_MOESM1_ESM.doc]

**FAM46C is critical for the anti-proliferation and pro-apoptotic effects of norcantharidin in hepatocellular carcinoma cells**

Qiao-Yan Zhang^1*^, Xiao-Qiang Yue^2*^, Yi-Ping Jiang^1^, Ting Han^1^, Hai-Liang Xin^1#^

^1^Department of Pharmacognosy, School of Pharmacy, Second Military Medical University, Shanghai 200433, P.R. China

^2^Department of Traditional Chinese Medicine, Changzheng Hospital, Second Military Medical University, Shanghai 200433, P.R. China

^*^Co-first author

^#^Correspondence author: Hai-Liang Xin, Department of Pharmacognosy, School of Pharmacy, Second Military Medical University. No. 325 Guohe Road, Shanghai 200433, P.R. China; Fax and telephone: (+86) 21-81871300, E-mail: hailiangxin00@163.com.

**Table S1.** Genes up-regulated by NCTD treatment.

**Table S2.** Genes down-regulated by NCTD treatment.


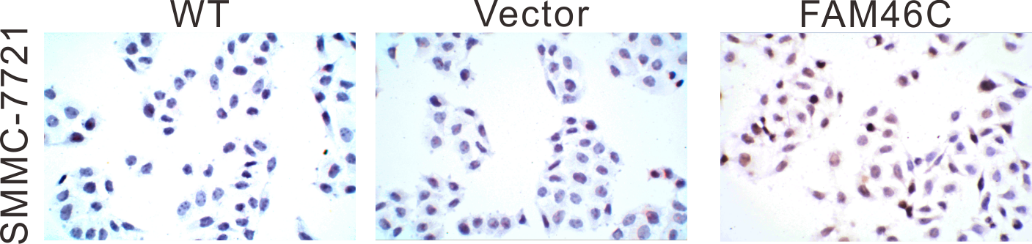


**Figure S1.** FAM46C overexpression resulted in a significant increase of cell apoptosis as determined by TdT-mediated-dUTP nick end labeling (TUNEL) Assays. SMMC-7721 cells cultured in coverslip were infected with Vector or FAM46C expressing lentivirus. At 48 h after infection, cells were fixed and subjected to TUNEL staining (brown) with *In Situ* Cell Death Detection Kit (Roche) according to the manufacturer’s protocol.

**
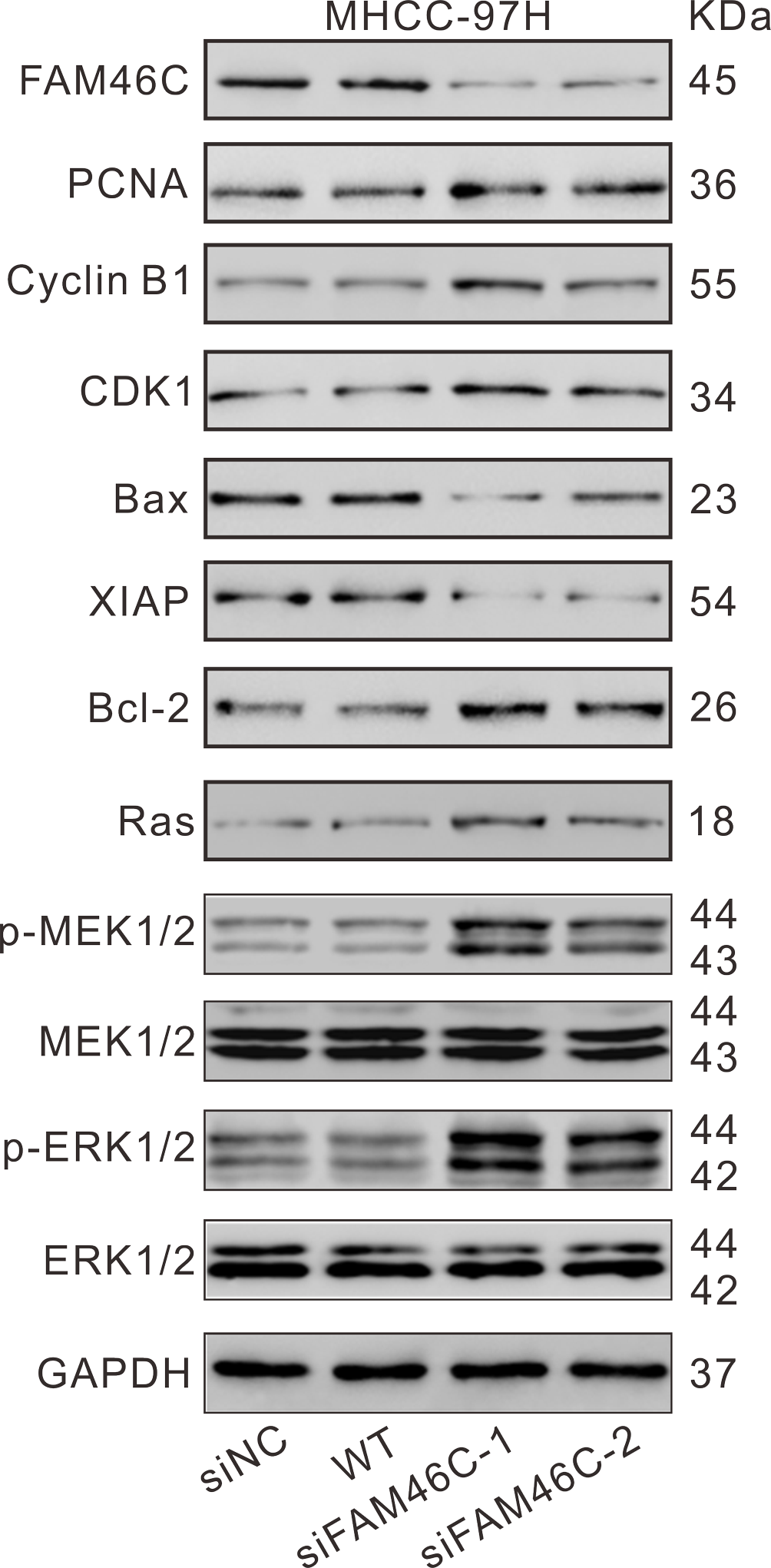
**

**Figure S2.** MHCC-97H cells were transfected with siNC, siFAM46C-1 or siFAM46C-2. At 48 h after transfection, the protein levels of FAM46C, and molecules related with cell cycle, cell apoptosis, and RAS/MEK/ERK were detected by Western blotting.

**
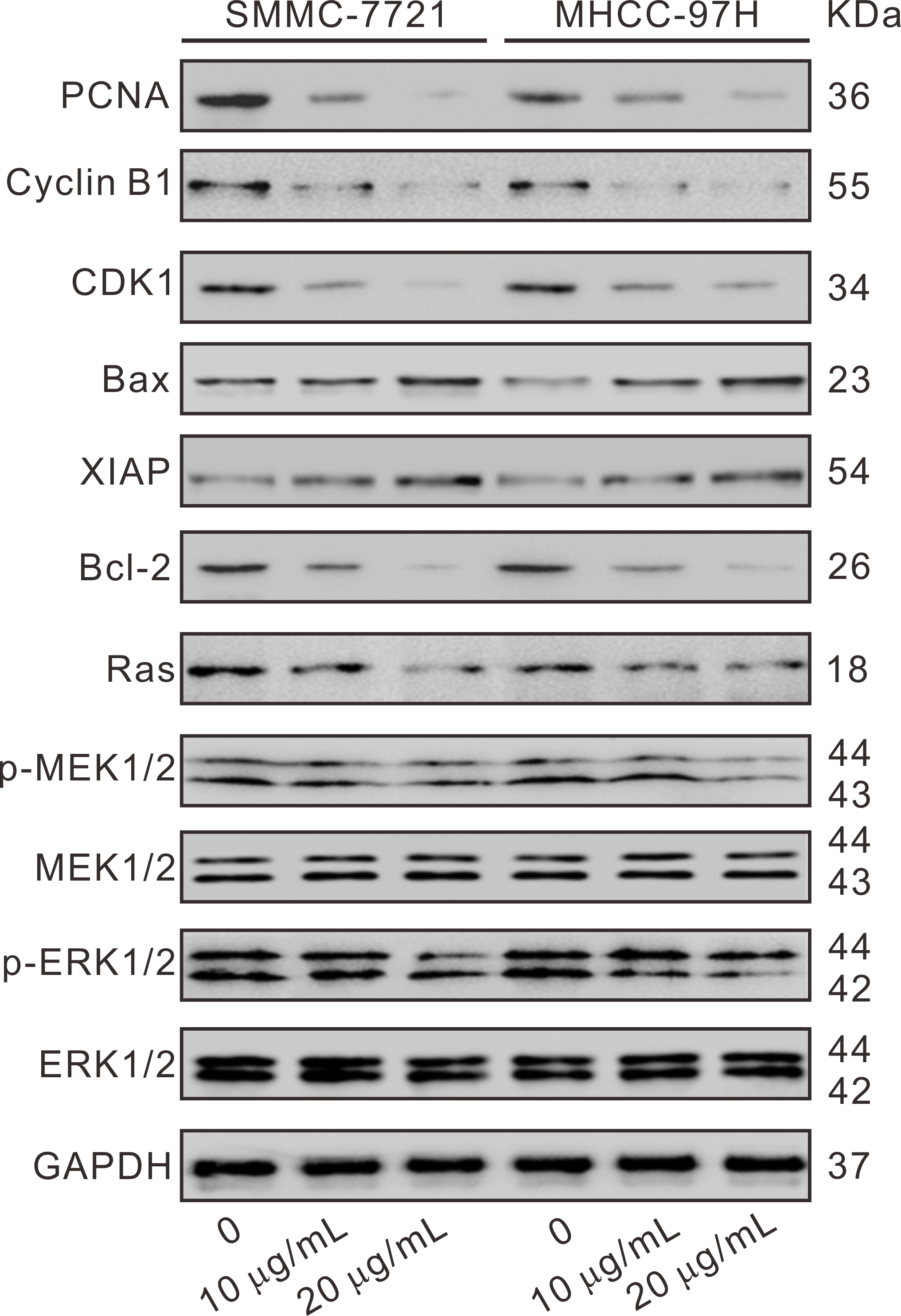
**

**Figure S3.** SMCC-7721 and MHCC-97H cells were treated with DMSO or NCTD (10 and 20 µg/mL) for 48 h. The protein levels of molecules related with cell cycle, cell apoptosis, and RAS/MEK/ERK were detected by Western blotting.
